# Retraction Note: Effect of Oroxylum indicum on hepatocellular carcinoma via the P53 and VEGF pathways based on microfluidic chips

**DOI:** 10.1186/s12906-025-04776-3

**Published:** 2025-01-25

**Authors:** Xi Luo, Miao Zhao, Sicong Liu, Yi Zheng, Qiang Zhang, Yong‑rui Bao, Shuai Wang, Tian‑jiao Li, Xian‑sheng Meng

**Affiliations:** 1 College of Pharmacy, Liaoning University of Traditional Chinese Medicine, Dalian, 116600 People’s Republic of China; 2 College of Integrated Chinese and Western Medicine, Liaoning University of Traditional Chinese Medicine, Shenyang, Liaoning, 110847 China; 3 Liaoning Multidimensional Analysis of Traditional Chinese Medicine Technical Innovation Center, Dalian, 116600 China; 4 Liaoning Province Modern Traditional Chinese Medicine Research and Engineering Laboratory, Dalian, 116600 China

**BMC Complementary Medicine and Therapies (2023) 23:400**

10.1186/s12906-023-04217-z

The Editor has retracted the article, following reports of image integrity concerns. Specifically, in Fig. 5 the images for 0h and IO, representing different experimental conditions, appear to substantially overlap. Similarly, Fig. 8a and 8d appear to be similar, despite also representing different experimental conditions. Finally, a number of artifacts appear to repeat within Fig. 8e.

The authors did not provide a satisfactory explanation and therefore the Editor no longer has confidence in the integrity of the data in this article.

Author Xi Luo disagrees with this retraction. All other authors did not respond to correspondence from the publisher regarding this retraction.

